# Voriconazole versus itraconazole for antifungal prophylaxis following allogeneic haematopoietic stem-cell transplantation

**DOI:** 10.1111/j.1365-2141.2011.08838.x

**Published:** 2011-11

**Authors:** David I Marks, Antonio Pagliuca, Christopher C Kibbler, Axel Glasmacher, Claus-Peter Heussel, Michal Kantecki, Paul JS Miller, Patricia Ribaud, Haran T Schlamm, Carlos Solano, Gordon Cook, IMPROVIT Study Group

**Affiliations:** 1 University Hospitals Bristol NHS Foundation TrustBristol, UK; 2 King's College HospitalLondon, UK; 3 Royal Free Hospital and University CollegeLondon, UK; 4 Rheinische Friedrich Wilhelms UniversitätBonn, Germany; 5 Radiology, Thoraxklinik at University HospitalHeidelberg, Germany, Germany; 6 Pfizer International OperationsParis, France; 7 Pfizer Global Research and DevelopmentSandwich, UK; 8 Hôpital Saint-LouisParis, France; 9 Pfizer IncNew York, NY, USA; 10 Hospital Clínico Universitario, University of ValenciaValencia, Spain; 11 Leeds Teaching HospitalsLeeds, UK

**Keywords:** stem-cell transplant, azoles, invasive fungal disease, mould infections, yeast infections

## Abstract

Antifungal prophylaxis for allogeneic haematopoietic stem-cell transplant (alloHCT) recipients should prevent invasive mould and yeast infections (IFIs) and be well tolerated. This prospective, randomized, open-label, multicentre study compared the efficacy and safety of voriconazole (234 patients) versus itraconazole (255 patients) in alloHCT recipients. The primary composite endpoint, success of prophylaxis, incorporated ability to tolerate study drug for ≥100 d (with ≤14 d interruption) with survival to day 180 without proven/probable IFI. Success of prophylaxis was significantly higher with voriconazole than itraconazole (48·7% vs. 33·2%, *P <*0·01); more voriconazole patients tolerated prophylaxis for 100 d (53·6% vs. 39·0%, *P*<0·01; median total duration 96 vs. 68 d). The most common (>10%) treatment-related adverse events were vomiting (16·6%), nausea (15·8%) and diarrhoea (10·4%) for itraconazole, and hepatotoxicity/liver function abnormality (12·9%) for voriconazole. More itraconazole patients received other systemic antifungals (41·9% vs. 29·9%, *P*<0·01). There was no difference in incidence of proven/probable IFI (1·3% vs. 2·1%) or survival to day 180 (81·9% vs. 80·9%) for voriconazole and itraconazole respectively. Voriconazole was superior to itraconazole as antifungal prophylaxis after alloHCT, based on differences in the primary composite endpoint. Voriconazole could be given for significantly longer durations, with less need for other systemic antifungals.

Invasive fungal infections (IFIs) are a major cause of morbidity and mortality after allogeneic haematopoietic stem-cell transplantation (alloHCT) (Ninin *et al*, 2001; Fukuda *et al*, 2003; Garcia-Vidal *et al*, 2008). Invasive aspergillosis (IA) is the most frequent IFI in this setting (1-year incidence of 11–14%) (Fukuda *et al*, 2003; Garcia-Vidal *et al*, 2008), with major risk factors including graft-versus-host disease (GvHD), the use of immunosuppressive drugs for GvHD and cytopenia (Garcia-Vidal *et al*, 2008; Mikulska *et al*, 2009). Mortality from invasive *Aspergillus* infections following alloHCT remains high – between 67% and 87% (Lin *et al*, 2001; Kojima *et al*, 2004; Mikulska *et al*, 2009) – though newer agents (including voriconazole) may have improved survival (Upton *et al*, 2007; Neofytos *et al*, 2009).

Because IFIs are difficult to diagnose and treat early, efforts have turned to prevention. Effective broad-spectrum (covering both moulds and yeasts) antifungal prophylaxis in alloHCT patients may reduce IFI incidence, morbidity and mortality (Bow *et al*, 2002; Fukuda *et al*, 2003). Oral azole antifungals have the potential to be more convenient and cost-effective in this setting. However, the optimal antifungal prophylaxis is unknown, and there is a need to identify more effective, better-tolerated agents. Fluconazole effectively prevents invasive candidiasis during the post-engraftment period (Goodman *et al*, 1992; Slavin *et al*, 1995), but does not have activity against *Aspergillus*. Itraconazole, a broad-spectrum azole also active against filamentous fungi, has shown efficacy in this setting (Glasmacher *et al*, 2003; Winston *et al*, 2003; Marr *et al*, 2004; Vardakas *et al*, 2005; Simon *et al*, 2007). However, the variable bioavailability of itraconazole tablets and poor tolerability of itraconazole suspension may limit its use as a prophylactic agent (Vardakas *et al*, 2005; Cornely *et al*, 2007; Simon *et al*, 2007). The second-generation triazole posaconazole also has anti-mould activity and was demonstrated to be effective as primary prophylaxis for specific alloHCT patients in a comparative trial with fluconazole (Ullmann *et al*, 2007). However, to date no mould-active agents have been compared head-to-head in this setting.

Voriconazole is a second-generation, broad-spectrum triazole with *in vitro* and clinical activity against yeasts and moulds, including *Aspergillus*, *Candida*, *Fusarium* and *Scedosporium* species, but not zygomycetes (Cecil & Wenzel, 2009). Voriconazole has demonstrated safety and efficacy as first-line treatment for invasive aspergillosis (Herbrecht *et al*, 2002) and as first-line treatment of serious *Candida* infections (Kullberg *et al*, 2005), and can be given as a bioavailable oral (Cecil & Wenzel, 2009) or an intravenous formulation. We evaluated the efficacy, safety and tolerability of voriconazole versus itraconazole as antifungal prophylaxis in alloHCT recipients, representing the first head-to-head comparison of two mould-active, orally available agents in this setting.

## Patients and methods

### Study design

This prospective, phase 3, randomized, open-label trial was conducted from March 2006 to February 2009 in 47 transplant centres across 12 countries, in compliance with the Declaration of Helsinki, International Conference on Harmonization Good Clinical Practice (GCP) guidelines and local regulatory requirements. All participants gave written informed consent. The protocol was approved by an institutional review board or independent ethics committee at each study site.

### Patients

Patients were aged ≥12 years and received sibling or unrelated donor alloHCT for acute leukaemia, myelodysplasia, transformed chronic myeloid leukaemia, or failed lymphoma therapy. Patients with myeloablative and reduced-intensity conditioning regimens were included. Patients with a probable/proven IFI during the 6 months prior to study entry, a history of zygomycosis, impaired hepatic function, or use of systemic antifungals within 7 d before study entry were excluded. Patients who received concomitant medications with major interactions with azoles were also not permitted to enter the study. Detailed inclusion and exclusion criteria are listed in the online data supplement (Data S1).

### Stratification and randomization

Patients were randomly assigned with equal probability to either voriconazole (Vfend; Pfizer Inc, New York, NY, USA) or itraconazole (Sporanox; Ortho-McNeil Janssen-Pharmaceuticals Inc, Raritan, NJ, USA) using a permuted block randomization (block size 4) with stratification by conditioning regimen (myeloablative or reduced-intensity) and donor relatedness (matched related or unrelated). Randomization was also blocked by centre.

Prophylaxis was scheduled to start on the day of alloHCT, at least 48 h after conditioning chemotherapy. The first day of study drug was considered as day 1. Following 1 d of intravenous loading (6 mg/kg every 12 h), voriconazole was administered as tablets or oral suspension at a dose of 200 mg twice daily; the dose was halved for patients <40 kg. Following 2 d of intravenous loading doses (200 mg every 12 h), itraconazole was given as oral solution at a dose of 200 mg twice daily. Itraconazole capsules were permitted for up to 14 d if patients were temporarily unable to continue oral solution. In case of mucositis or gut GvHD, patients could be given either study drug intravenously (voriconazole: 4 mg/kg twice daily; itraconazole: 200 mg once daily). Prophylaxis with study drug was to be given for ≥100 d for all patients and could be extended to day 180 if risk factors for IFI persisted (Data S1). Regardless of study drug duration, all patients were followed for 180 d for development of IFIs and 1 year for survival.

Systemic antifungal therapy with a non-study agent could be initiated for up to 14 d for persistent fever or signs of possible IFI (Ascioglu *et al*, 2002) pending confirmation of a proven/probable IFI, at the discretion of the investigator, without the patient being classified a prophylactic failure. Given that both study drugs have activity against *Aspergillus* and because testing was not universally available, a structured IFI screening programme with galactomannan testing was not employed. An independent, blinded data review committee reviewed all suspected and documented IFIs that occurred during the study period and categorized them according to consensus criteria current at study onset (Data S1) (Ascioglu *et al*, 2002).

### Endpoints

In light of the fact that previous studies comparing different agents as antifungal prophylaxis post-alloHCT were unable to show any significant differences in the overall incidence of IFI or in patient survival, a composite endpoint was chosen for the purposes of this trial. The primary endpoint, success of prophylaxis, was defined as the ability to tolerate study drug for at least 100 d, with ≤14 d interruption, with survival without proven/probable IFI to day 180. All patients who discontinued study drug for more than 14 d in the 100-d prophylaxis period, who died before or on day 180, or were diagnosed with a proven/probable IFI before or on day 180 were regarded as treatment failures. Secondary analyses included comparison of success of prophylaxis at day 100, proven/probable IFI, use of systemic antifungal agents and survival to day 180 and 1 year. Treatment satisfaction was patient-assessed using a modified Treatment Satisfaction Questionnaire for Medication (TSQM) and compared at day 14 (Atkinson *et al*, 2004, 2005). All analyses were conducted in the modified intent-to-treat (mITT) population, which included all randomized patients who had given informed consent, received alloHCT and received at least one dose of study medication. In addition, it was planned to assess plasma levels of study drug on day 14 and at the time of breakthrough IFI, using standard methods (Srivatsan *et al*, 2004; Andrews *et al*, 2008); itraconazole plasma levels were also to be evaluated after capsule use.

### Safety assessment

Standard haematological and biochemical laboratory tests were performed at screening and on days 0, 2, 14, 28, 56, 100, 140 and 180, while patients were receiving study drug. Electrocardiography was performed at screening and on days 2 and 28. Adverse events and serious adverse events were reported until 14 and 28 d after last dose of study drug respectively. Adverse event causality was assessed by investigators.

### Statistical analysis

The primary analysis of the trial was intended to demonstrate the non-inferiority of voriconazole to itraconazole in the comparison of success of prophylaxis at day 180 in the mITT population. Non-inferiority was inferred if the lower limit of the two-sided 95% confidence interval (CI) for the difference in adjusted success rates (Data S1) at day 180 was ≥10%. If non-inferiority was demonstrated, superiority would be inferred if this two-sided 95% CI was positive. Assuming success rates of 50% for voriconazole and 45% for itraconazole, a sample size of 232 patients per group has 90% power to demonstrate non-inferiority of voriconazole to itraconazole, and ≥80% power to demonstrate superiority of voriconazole if the true success rates for voriconazole and itraconazole were 57% and 44% respectively. *P* values < 0·05 were considered significant.

## Results

### Study population

**Fig 1:**
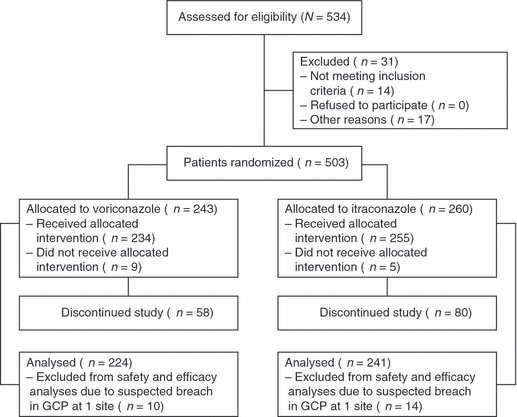
Patient CONSORT flow chart. GCP, Good Clinical Practice.

A total of 534 patients were screened, 503 were randomized, and 489 received at least one dose of study medication (voriconazole *n*=234, itraconazole *n*=255; Fig 1). Detailed reasons for the exclusion of screened patients from randomization, and why some randomized patients did not receive study treatment, are listed in Data S1. Due to a suspected breach in GCP, all patients from one study site (10 received voriconazole, 14 received itraconazole) were excluded from all analyses. Baseline characteristics, including conditioning regimen and underlying haematological condition, were well matched between the two arms (Table I). Treatment groups were also balanced in the proportion of patients developing GvHD (Data S1) and undergoing T-cell depletion.

**Table I:** Patient baseline characteristics for the modified intent-to-treat population*

	Voriconazole (*n*=224)	Itraconazole (*n*=241)
Randomization stratum, *n* (%)		
Myeloablative and matched related	66 (29·5)	85 (35·3)
Myeloablative and mismatched/unrelated	59 (26·3)	58 (24·1)
Non-myeloablative and matched related	58 (25·9)	57 (23·7)
Non-myeloablative and mismatched/unrelated	41 (18·3)	41 (17·0)
Primary diagnosis, *n* (%)		
Acute lymphocytic leukaemia	41 (18·3)	41 (17·0)
Acute myeloid leukaemia	98 (43·8)	109 (45·2)
Myelodysplastic syndrome	34 (15·2)	30 (12·4)
Failure of therapy for lymphoma	42 (18·8)	46 (19·1)
Transformation of chronic myeloid leukaemia	6 (2·7)	13 (5·4)
Other†	3 (1·3)	2 (0·8)
Conditioning regimen, *n* (%)‡		
Myeloablative	125 (55·8)	143 (59·3)
Non-myeloablative	99 (44·2)	98 (40·7)
Sex, *n* (%)		
Male	130 (58·0)	146 (60·6)
Female	94 (42·0)	95 (39·4)
Age, years		
Mean	43·3	42·3
Range	11–70	13–70
Ethnicity, *n* (%)		
White	207 (92·4)	219 (90·9)
Black	0 (0·0)	2 (0·8)
Asian	2 (0·9)	3 (1·2)
Other	15 (6·7)	17 (7·1)
Body mass index, kg/m^2^		
Mean	25·5	25·8
Range	15·7–41·8	14·9–49·5

* The modified intent-to-treat population included all patients who underwent haematopoietic stem-cell transplant and received at least one dose of study drug. Patients from one study site were excluded due to a suspected Good Clinical Practice breach.

† Primary diagnoses not permitted by the study protocol, i.e. myeloma (two voriconazole patients) and chronic lymphocytic leukaemia (one voriconazole patient, two itraconazole patients).

‡ 34·4% of voriconazole patients and 33·6% of itraconazole patients underwent *in vivo* T-cell depletion, i.e. received antithymocyte immunoglobulin and/or alemtuzumab prior to screening (*P*=0·86).

### Efficacy

Success of antifungal prophylaxis at day 180, the primary endpoint, was demonstrated in 48·7% of voriconazole and 33·2% of itraconazole patients, a difference of 16·4% (95% CI, 7·7–25·1; *P*=0·0002) after adjustment for randomization strata. At day 100, the adjusted difference in success of prophylaxis was 15·4% (95% CI, 6·6–24·2; *P*<0·01), favouring voriconazole (54·0% vs. 39·8% respectively). The difference in success rates between treatments did not vary across randomization strata (day 100, *P*=0·29; day 180, *P*=0·41).

The proportion of patients who completed ≥100 d of study drug prophylaxis was 53·6% for voriconazole versus 39·0% for itraconazole (95% CI of difference, 5·6–23·5; *P*<0·01). Median total durations of study drug treatment were 96 and 68 d respectively (*P*<0·01). After the initial intravenous dosing period, 112 (46·5%) itraconazole patients and 83 (37·1%) voriconazole patients received intravenous study drug for at least 1 d (95% CI of difference, 0·5–18·3; *P*=0·04). Median durations of intravenous treatment were 10 and 11 d respectively. Thirty-four (14·1%) itraconazole patients received capsules for at least 1 d, with a mean duration of 11·7 d.

**Fig 2:**
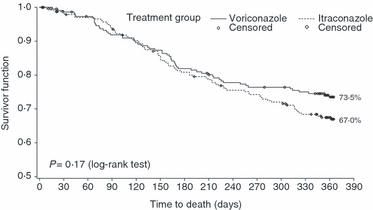
Kaplan–Meier survival estimates from start of prophylaxis until day 365 for patients treated with voriconazole and itraconazole.

Kaplan–Meier estimates of survival at day 100 (91·9% for voriconazole, 92·3% for itraconazole) and day 180 (81·9% for voriconazole, 80·9% for itraconazole) were similar. One-year survival rates were 73·5% and 67·0% for voriconazole and itraconazole respectively (*P*=0·17; log-rank test). The hazard ratio for death in the voriconazole group compared with the itraconazole group was 0·79 (95% CI, 0·56–1·11). Kaplan–Meier survival estimates from start of prophylaxis until day 365 by treatment are presented in Fig 2.

A total of three (1·3%) voriconazole patients developed a proven or probable IFI during the study period, compared with five (2·1%) itraconazole patients (95% CI for difference, −3·1 to 1·6; *P*=0·54; Table II). These IFIs occurred earlier with itraconazole than voriconazole (average time to IFI: 73·8 vs. 118·0 d) and the only two treatment-emergent IFIs (defined as IFIs while receiving study drug or within 7 d of discontinuation) occurred in patients receiving itraconazole. There were slightly more documented *Aspergillus* infections reported in itraconazole patients (five vs. one respectively; *P*=0·12), but only one, in an itraconazole patient, was fatal. There were no cases of zygomycosis reported in either study arm.

**Table II:** Invasive fungal infections during the study (diagnosed according to EORTC/MSG criteria; Ascioglu *et al*, 2002)

Level of diagnosis	Pathogen	Body site of IFI	Last dose of study drug (d)	Onset of IFI (d)
Voriconazole arm				
Proven	*Candida krusei*	Blood	27	100
Proven	*Candida parapsilosis*	Blood	104	151
Probable	*Aspergillus fumigatus*	Lung	48	103
Itraconazole arm				
Proven	*Aspergillus fumigatus*	Lung	19	82
Probable	*Aspergillus* spp.	Lung	9	11
Probable	*Aspergillus* spp.	Lung	21	20
Probable	*Aspergillus fumigatus*	Lung	14	80
Probable	*Aspergillus* spp.	Lung	20	176

EORTC/MSG, European Organization for Research and Treatment of Cancer/Mycoses Study Group; IFI, invasive fungal infection.

### Treatment satisfaction

TSQM data were available for the majority of patients on day 14; the proportions of patients with these data were similar for both treatments (Data S1). Based on these data, voriconazole was superior to itraconazole in effectiveness (74·5 vs. 67·9; *P*<0·01), convenience (75·3 vs. 65·0; *P*<0·01) and global satisfaction (70·6 vs. 63·1; *P*<0·01). Both study treatments were similar in side-effect scores (91·7 vs. 88·4; *P*=0·17). The global satisfaction score at day 14 was a significant predictor of the ability to complete 100 d of prophylaxis (*P*=0·02 on Cox regression).

### Plasma levels

Plasma drug levels at steady state were available in 116 voriconazole and 130 itraconazole patients (51·8% and 53·9% respectively). However, only 34 voriconazole patients (15·2%) had trough levels with a median concentration of 0·85 μg/ml (range 0–4·53 μg/ml). Trough levels were >0·5 and >1 μg/ml in 22 (64·7%) and 13 (38·2%) of these patients respectively. In itraconazole patients, 24 patients (10·0%) had trough levels with a median concentration of 0·89 μg/ml (range 0–2·46 μg/ml). Trough levels were >0·5 and >1 μg/ml in 20 (83·3%) and 11 (45·8%) of these patients respectively.

### Safety and tolerability

The most frequent all-causality adverse events in both treatment arms were mucosal inflammation, diarrhoea, pyrexia, vomiting and nausea. A higher incidence of serious all-causality adverse events occurred with voriconazole (47·8% vs. 37·3%, *P* = 0·02), but the period of observation was substantially longer.

Treatment-related gastrointestinal side effects (nausea, vomiting and diarrhoea) were more common with itraconazole (*P*<0·01 for each; Table III). Treatment-related hepatotoxicity/liver function abnormalities occurred more frequently in voriconazole patients (12·9% vs. 5·0%, *P*<0·01), and five were graded as severe, compared with one in the itraconazole arm (*P*=0·08). When adjusted for duration of observation, the average number of treatment-related adverse events per 30 d of treatment was 1·7 (95% CI, 1·1–2·2) for voriconazole and 2·0 (95% CI, 1·3–2·6) for itraconazole (*P*=0·53). Of the five voriconazole patients with severe hepatotoxicity, four survived to the 1-year follow-up visit and none were considered by the investigators to have died of study drug-related causes. Visual impairment was also more frequent with voriconazole (5·4% vs. 0·0%; *P*<0·01), but all cases of visual impairment with voriconazole were mild to moderate in severity, non-serious and resolved without sequelae.

**Table III:** Most common treatment-related adverse events (≥5% in either group) among modified intent-to-treat patients.

Adverse event	Voriconazole (*n*=224) *n* (%)	Itraconazole (*n*=241) *n* (%)	*P* value
Vomiting	8 (3·6)	40 (16·6)	<0·01
Nausea	16 (7·1)	38 (15·8)	<0·01
Diarrhoea	9 (4·0)	25 (10·4)	<0·01
Hepatotoxicity/liver function test abnormality	29 (12·9)	12 (5·0)	<0·01
Headache	10 (4·5)	12 (5·0)	0·79
Visual impairment	12 (5·4)	0 (0)	<0·01

Randomized study treatment was discontinued prior to day 100 in 147 itraconazole compared with 104 voriconazole patients (61·0% vs. 46·4%; *P* < 0·01). The most common investigator-assessed reasons for itraconazole discontinuation were adverse events (23·2%) and study drug intolerance (21·6%). The most common reason for voriconazole discontinuation was adverse events (29·9%; Data S1).

### Use of other systemic antifungal agents

At least one systemic antifungal agent other than randomized study drug was given during the study period in 101 itraconazole patients and 67 voriconazole patients (41·9% vs. 29·9%; *P*<0·01). Forty-three (17·8%) itraconazole and 11 (4·9%) voriconazole patients received more than one such agent (*P*<0·01). More itraconazole patients received liposomal amphotericin B and/or caspofungin (23·2% versus 15·2%, *P*=0·03), with respective median durations of 14 versus 10 d (*P*=0·19). Thirty-seven (15·4%) itraconazole patients received voriconazole and/or posaconazole, and 10 (4·5%) voriconazole patients received itraconazole or posaconazole (Table IV).

**Table IV:** Other systemic antifungal agents given during the study period*

Systemic antifungal agent	Voriconazole (*n*=224) *n* (%)	Itraconazole (*n*=241) *n* (%)	*P* value
Any systemic antifungal agent	67 (29·9)	101 (41·9)	<0·01
Caspofungin	24 (10·7)	48 (19·9)	<0·01
Liposomal amphotericin B	14 (6·3)	17 (7·1)	0·73
Caspofungin and/or liposomal amphotericin B	34 (15·2)	56 (23·2)	0·03
Amphotericin B†	4 (1·8)	7 (2·9)	0·43
Fluconazole	21 (9·4)	37 (15·4)	0·051
Itraconazole‡	5 (2·2)	8 (3·3)	0·48
Voriconazole‡	9 (4·0)	34 (14·1)	<0·01
Posaconazole‡	5 (2·2)	11 (4·6)	0·17

* Substantial numbers of patients received more than one such agent: 43 (17·8%) itraconazole and 11 (4·9%) voriconazole patients.

† In addition, 31 patients (30 from one site) received aerosolized amphotericin B during the study: 16 voriconazole patients and 15 itraconazole patients.

‡ Ten voriconazole patients (4·5%) received itraconazole or posaconazole. 37 itraconazole patients (15·4%) received voriconazole and/or posaconazole. Some patients who discontinued study therapy subsequently recommenced the agent they were originally randomized to; this was recorded as other licensed antifungal therapy.

## Discussion

This large, randomized trial represents the first direct, prospective comparison of two mould-active, orally available agents as antifungal prophylaxis after alloHCT. Based on its superiority in the composite primary endpoint incorporating tolerability, IFI prevention and survival, voriconazole was shown to be more effective than itraconazole for antifungal prophylaxis in this setting. The main driver for this difference was that significantly more voriconazole patients were able to tolerate at least 100 d of study drug with minimal interruption.

In this study we compared voriconazole with itraconazole, another mould-active antifungal agent. Due to the high risk for IA in this population (Fukuda *et al*, 2003; Garcia-Vidal *et al*, 2008), it was important to implement a study comparing two mould-active agents for appropriate antifungal prophylaxis, given that such an evaluation had not been prospectively conducted to date. Because both agents have the potential to prevent IFI, including *Aspergillus* infections, the ability to tolerate study drug for relatively long durations becomes an important consideration. In fact, current transplant regimens are associated with prolonged periods of immunosuppression, and IFIs (particularly IA) may develop for up to 6 months after alloHCT (Garcia-Vidal *et al*, 2008). In this study, voriconazole was better tolerated than itraconazole for longer durations. The major treatment-limiting side effects of itraconazole were related to gastrointestinal intolerance, including nausea, vomiting and diarrhoea.

Despite the higher incidence of treatment-related hepatic and visual adverse events reported with voriconazole, patients were able to continue voriconazole for longer periods than itraconazole. The overall safety profile for voriconazole in this study was consistent with previous reports in similar patient populations (Herbrecht *et al*, 2002; Queiroz-Telles *et al*, 2007; Cecil & Wenzel, 2009). For example, a recently published noncomparative study of voriconazole as secondary prophylaxis in allograft recipients reported hepatotoxicity in 4/45 (9%) patients; treatment duration was similar to that in our trial (Cordonnier *et al*, 2010). The higher rates of hepatotoxicity seen in the voriconazole arm (13% vs. 5%) need to be considered in the context of the patient population. The majority of allograft patients experience disturbances in hepatic function, which are commonly multifactorial in origin (e.g. due to GvHD or concomitant medications); this makes it difficult to attribute abnormal liver function tests specifically to one drug or medical condition. Notably, significant derangement of hepatic function during the early post-transplant phase can be an issue that requires adjustment of prescribed drugs, including calcineurin inhibitors. Of the five voriconazole patients (compared with one itraconazole patient) with severe hepatotoxicity, four survived to the 1-year follow-up visit, suggesting that these liver function test abnormalities were generally reversible.

The better tolerability of voriconazole compared with itraconazole was reflected in the TSQM results: patients receiving voriconazole reported higher convenience and global satisfaction scores at 2 weeks after start of study treatment. The latter score correlated with the ability of voriconazole patients to complete at least 100 d of study drug prophylaxis.

In terms of IFI prevention and overall survival, there were no statistically significant differences between voriconazole and itraconazole. However, it should be noted that voriconazole patients required significantly fewer other licensed systemic antifungal agents, including caspofungin and liposomal amphotericin B. These findings are mirrored in a number of other recently published azole prophylaxis trials in the same setting. For instance, a randomized, double-blind study comparing voriconazole with fluconazole in standard-risk alloHCT recipients [conducted by the Blood and Marrow Transplant Clinical Trials Network (BMT-CTN)] was also unable to show differences in IFI incidence or overall survival, but similarly reported a lower use of empirical antifungal therapy in voriconazole patients (Wingard *et al*, 2010). Of note, another randomized trial, evaluating posaconazole against fluconazole in high-risk alloHCT recipients, also failed to demonstrate a significant difference in overall IFI incidence or survival, but reported fewer cases of proven or probable IA in the posaconazole arm (Ullmann *et al*, 2007). On the other hand, in a small retrospective study conducted in a similar population, voriconazole was more effective than fluconazole/itraconazole in preventing not just IA, but also IFIs overall (Gergis *et al*, 2010). The lack of significant differences in IFI incidence or survival during previous prospective clinical trials prompted us to choose a composite measure as the primary endpoint in this study, in order to facilitate the detection of relevant clinical differences between the two study drugs. Similar composite endpoints may also be useful in future comparative trials in antifungal prophylaxis.

Of note, the incidence of breakthrough IFIs in our trial was unusually low compared with other published studies. One possible explanation is that it was not always possible to perform bronchoscopy or biopsy for the purpose of confirming invasive fungal disease in this patient population. In addition, routine galactomannan monitoring was not part of our study design. In contrast, the BMT-CTN study did incorporate intensive galactomannan monitoring, which facilitated the diagnosis of more than half of all probable IA cases in that trial (Wingard, *et al*, 2010). However, the value of routine galactomannan screening in a study comparing mould-active agents for prophylaxis is debatable (Marr *et al*, 2005). We used the 2002 European Organization for Research and Treatment of Cancer/Mycoses Study Group (EORTC/MSG) definitions of proven or probable IFI in our study (Ascioglu *et al*, 2002), but the utility of these definitions in the context of antifungal prophylaxis trials has recently been questioned (Wingard *et al*, 2010). For example, the BMT-CTN study incorporated a new category of ‘presumptive IFI’ (Wingard *et al*, 2010). We are planning a future analysis of breakthrough invasive fungal disease in our study, which will include possible, in addition to probable and proven, IFIs based on the latest EORTC/MSG definitions.

Our findings may also have been affected by the inclusion of patients with reduced-intensity conditioning regimens, who were excluded from previous studies of antifungal prophylaxis after alloHCT. This population, which constitutes a major proportion of patients in modern transplant practice, may have a lower IFI risk particularly during the pre-engraftment period, but a similar risk of IFI (i.e. mainly IA) after engraftment (Martino *et al*, 2001, 2002).

Our study has some limitations. In theory, it would have been preferable to employ a blinded study design. The lack of blinding potentially affected investigator-assessed toxicities and decisions regarding the use of other antifungal agents. However, this approach would have required that voriconazole patients take an oral cyclodextrin placebo. Not only would this be unethical in patients who already have difficulties taking oral drugs, but it would also have impaired our ability to compare tolerability between the study agents.

Another limitation of the study is the lack of plasma drug level data in many patients: documented steady-state trough levels were available for only 15% of voriconazole and 10% of itraconazole patients. Among these, 83% of itraconazole patients had drug levels >0·5 μg/ml, which was previously recommended as the minimal serum concentration for this drug (Glasmacher *et al*, 2003). The target voriconazole concentration for prophylaxis is unknown; however, levels were >0·5 μg/ml in 65% and >1·0 μg/ml in 38% of patients with trough concentrations measured. There were insufficient data in this study to assess the relationship between voriconazole concentrations and efficacy or toxicity. Finally, it should be pointed out that the intravenous formulation of itraconazole is no longer commercially available; however, this should not be an issue in terms of extending our results to clinical practice, as few of our patients received intravenous itraconazole.

In conclusion, this study demonstrated that voriconazole and itraconazole were equivalent in terms of survival and prevention of IFI when used as antifungal prophylaxis after alloHCT. However, patients were able to receive voriconazole for significantly longer durations, despite the fact that more hepatic and visual toxicities were reported with this agent. In addition, there was less need for other systemic antifungals compared with itraconazole. In alloHCT recipients requiring a mould-active, orally available agent for the prevention of IFI, voriconazole may be a better option than itraconazole.

## Appendix

In addition to the authors, the following investigators participated in the IMPROVIT study: M. Abecasis, Instituto Português de Oncologia de Lisboa, Lisbon, Portugal; B. Afanasyev, Pavlov State Medical University, St. Petersburg, Russian Federation; H. Akan, Cebeci Hospital, Ankara, Turkey; A. Anagnostopoulos, Papanikolaou General Hospital of Thessaloniki, Thessaloniki, Greece; M. Batlle, Hospital Universitari Germans Trias i Pujol, Badalona, Spain; R.M. Bofarull, Hospital de la Santa Creu i Sant Pau, Barcelona, Spain; P. Bordigoni, Hôpital d'Enfants de Brabois, Vandoeuvre-lès-Nancy, France; J.-H. Bourhis, Institut Gustave-Roussy, Villejuif, France; E. Bow, Health Sciences Center, Manitoba, Canada; P. Cetkovsky, Institute of Haematology and Blood Transfusion, Prague, Czech Republic; C. Cordonnier, CHU Henri-Modor, Créteil, France; C. Crawley, Nuffield Health Cambridge Hospital, Cambridge, UK; R. de la Camara, Hospital de la Princesa, Madrid, Spain; B. Eser, Erciyes University Medical Faculty, Kayseri, Turkey; E. Espigado, Hospital Universitario Virgen del Rocio, Sevilla, Spain; A. El Hadad, Nasser Institute for Research and Treatment, Shubra, Egypt; J. de la Serna, Hospital 12 de Octubre, Madrid, Spain; C. Faucher, Institut Paoli Calmettes, Marseille, France; A. Gratwohl, Universitätsspital Basel, Basel, Switzerland; S. Haider, McMaster University Medical Centre, Hamilton, Canada; A. Hunter, Leicester Royal Infirmary, Leicester, UK; M. Larverdière, Hôpital Maisonneuve-Rosemont, Montréal, Canada; S. Leprêtre, Centre Henri Becquerel, Rouen, France; E. Liakopoulou. Christie Hospital, Manchester, UK; A. Maschan, Research Institute for Paediatric Haematology of the Russian Federation, Moscow, Russian Federation; J. Mayer, Fakultni Nemocnice Brno, Brno, Czech Republic; N. Milpied, Hôpital Haut-Lévêque, Pessac Bersol, France; S. Narayanan, Vancouver General Hospital, Vancouver, Canada; A. Parker, Glasgow Royal Infirmary, Glasgow, UK; J. Passweg, Hôpital Cantonal Universitaire de Genève, Geneva, Switzerland; J. Perez, Hospital Universitario Ramon y Cajal, Madrid, Spain; I. Peristeri, Aghia Sophia Children's Hospital, Athens, Greece; P. Pimentel, Instituto Português de Oncologia do Porto, Porto, Portugal; M. Potter, Royal Marsden Hospital, London, UK; O. Reman, CHU Caen Hôpital Clémenceau, Caen, France; M. Rovira, Hospital Clinic I Provincial de Barcelona, Barcelona, Spain; M. Saad, King Hussein Cancer Centre, Amman, Jordan; M. Sanz, Hospital Universitario La Fe, Valencia, Spain; M. Smith, Guy's Hospital, London, UK; L. Vazquez, Hospital Universitario de Salamanca, Salamanca, Spain; K. Wilson, Cardiff University School of Medicine, Cardiff, UK.

## Supporting information

**Data S1.** Additional information on Methods (inclusion/exclusion criteria, definition of proven and probable IFI, further details on statistical analyses) and Results (reasons for exclusion, GvHD and T-cell depletion, TSQM, reasons for study discontinuation).
