# Supplementary material for: Voriconazole versus itraconazole for antifungal prophylaxis following allogeneic haematopoietic stem-cell transplantation
Source: Br J Haematol. 2011 Nov;155(3):318–27. doi: 10.1111/j.1365-2141.2011.08838.x (PMC3253339; doi:10.1111/j.1365-2141.2011.08838.x)
Supplement: Supplementary file 1 [file bjh0155-0318-SD1.pdf]

ONLINE DATA SUPPLEMENT

Details of the inclusion criteria

Patients were required to meet all of the following inclusion criteria in order to be eligible for enrolment into the trial:

- Male or female, aged ≥12 years.
- Receive allogeneic haematopoietic stem-cell transplant (HCT) for acute leukaemia (acute myeloblastic leukaemia, acute lymphoblastic leukaemia, or myelodysplastic syndrome), failure of therapy for lymphoma, or transformation of chronic myeloid leukaemia.
- Provide signed and dated written informed consent in accordance with all local regulatory and legal requirements.
- In the case of females of childbearing potential: a negative serum beta-human chorionic gonadotrophin pregnancy test at the screening visit and use of an approved method of contraception throughout the study.

Details of the exclusion criteria

Patients presenting with any of the following were not included in the trial:

- Possible, probable, or proven invasive fungal infection (IFI) at study entry or at any time within 6 months prior to study entry, defined according to the European Organisation for Research and Treatment of Cancer/Mycoses Study Group (EORTC/MSG) consensus criteria from 2002 (Ascioglu, Rex *et al*, 2002).

- Previous history of invasive zygomycosis (e.g. due to *Mucor* spp, *Absidia* spp, or *Rhizopus* spp).
- Use of any systemically active antifungal agent within 7 days prior to study entry.
- Allergy to study drugs or any of the excipients.
- Impaired hepatic function, defined as transaminases or alkaline phosphatase greater than five times the upper limit of normal or total bilirubin >2·5 mg/dl.
- Severe disease, other than underlying condition, considered likely to jeopardise the planned termination of the study (e.g. acute myocardial infarction, unstable angina pectoris, or potentially pro-arrhythmic conditions such as cardiac impairment due to previous cardiotoxic chemotherapy, previous *torsade de pointes*, or prolongation of the QT interval >450 ms for men or >470 ms for women).
- Concomitant use of sirolimus, ergot alkaloids, terfenadine, astemizole, cisapride, pimozide, quinidine, carbamazepine, rifampicin, phenobarbital, ritonavir, efavirenz, or St. John's wort.
- Alcohol and/or any other drug abuse.
- Previous participation in this trial.
- Unable and/or unlikely to comprehend and/or follow the protocol.
- Participation in any other studies involving investigational products, concomitantly or within 30 days prior to entry into this trial.
- Anticipated survival less than 1 month.

Conditions for continuation of prophylaxis beyond day 100

- Patient received prednisone (>0·2 mg/kg), muromonab-CD3, mycophenolate mofetil, infliximab, daclizumab, or alemtuzumab.
- Patient received antithymocyte globulin (ATG) or had received ATG within the 4 weeks prior to day 100.
- Patient was neutropaenic (absolute neutrophil count <500/mm^3) or lymphopaenic (absolute lymphocyte count <500/mm^3) or had been neutropaenic/lymphopaenic within the 10 days prior to day 100.
- Patient experienced graft-versus-host disease (GvHD) or another cause of immunosuppression expected to prolong the risk for developing an IFI beyond day 100.

Note: If a patient completed prophylaxis to day 100 and stopped study drug, it could be restarted under these same conditions.

Definition of probable or proven IFIs

Proven or probable IFIs were defined according to the EORTC/MSG consensus criteria from 2002 (Ascioglu, Rex *et al*, 2002).

Proven IFI

A proven IFI was confirmed in case the patient met one of the following criteria:

- Histopathology or cytopathology showing hyphae from needle aspiration or biopsy with evidence of associated tissue damage.

- Histopathology or cytopathology showing yeast cells and/or pseudohyphae from a needle aspiration or biopsy (excluding mucous membranes).
- Positive culture obtained from a normally sterile and clinically or radiologically abnormal site consistent with infection (excluding urine and mucous membranes in the case of moulds and sinuses, urine and mucous membranes in the case of yeasts).
- Blood culture positive for fungi (excluding *Aspergillus* spp and *Penicillium* spp other than *P marneffei*) along with temporally related clinical signs and symptoms compatible with the identified organism.
- Microscopy or antigen positivity for *Cryptococcus* spp in cerebrospinal fluid.

Probable IFI

A probable IFI was confirmed in case the patient met at least one of the following host criteria, one of the following microbiologic criteria and one of the following major (or two minor) clinical criteria:

Host criteria

- Neutropaenia (absolute neutrophil count <500/mm^3 for >10 days).
- Persistent fever for >96 hours refractory to broad-spectrum antibiotics in high-risk patients.
- Body temperature either >38°C or <36°C and any of the following predisposing conditions:
  - Prolonged (>10 days) neutropaenia during the previous 60 days.

    - Current or recent (previous 30 days) use of significant immunosuppressive agents.
    - Previous proven or probable IFI during episode of neutropaenia.
    - Symptomatic AIDS.
- Presence of GvHD, particularly severe or chronic extensive GvHD.
- Prolonged (>3 weeks) use of corticosteroids during the previous 60 days.

Microbiologic criteria

- Positive culture for mould or *Cryptococcus neoformans* or an endemic fungal pathogen from sputum or bronchoalveolar lavage fluid samples.
- Positive culture or findings of cytological/direct microscopic evaluation for mould from sinus aspirate sample.
- Cytological/direct microscopic evaluation of sputum or bronchoalveolar lavage fluid samples positive for mould or *Cryptococcus* spp.
- Positive result for *Aspergillus* antigen in specimens of bronchoalveolar lavage fluid, cerebral spinal fluid (CSF), or at least two blood samples.
- Blood sample positive for cryptococcal antigen.
- Cytological/direct microscopic examination positive for fungal elements in sterile body fluid samples.
- Positive result for *Histoplasma capsulatum* antigen in blood, urine, or CSF samples.
- Two urine samples positive for yeasts in absence of urinary catheter.
- *Candida* casts in urine in absence of urinary catheter.
- Blood culture positive for *Candida* spp.

Clinical criteria

- Lower respiratory tract infection:
  - Major: Any of the following new infiltrates on computed tomography (CT) imaging: halo sign, air-crescent sign, or cavity within an area of consolidation.
  - Minor:
    - Symptoms of lower respiratory tract infection (cough, chest pain, haemoptysis, dyspnoea).
    - Pleural rub.
    - Any new infiltrate not fulfilling the major criterion.
    - Pleural effusion.
- Sinonasal infection:
  - Major: Suggestive radiologic evidence of invasive infection in sinuses (i.e. erosion of sinus walls or extension of infection to neighbouring structures, extensive skull base destruction).
  - Minor:
    - Upper respiratory symptoms (e.g. nasal discharge, stuffiness).
    - Nose ulceration or eschar of nasal mucosa or epistaxis.
    - Periorbital swelling.
    - Maxillary tenderness.
    - Black necrotic lesions or perforation of hard palate.

- Central nervous system (CNS) infection:
    - Major: Radiologic evidence suggestive of CNS infection (e.g. mastoiditis or other parameningeal foci, extradural empyema, intraparenchymal brain, or spinal cord mass lesion).
    - Minor:
        - Focal neurologic symptoms and signs (including focal seizures, haemiparesis and cranial nerve palsies).
        - Mental changes.
        - Meningeal irritation findings.
        - Abnormalities in CSF biochemistry and cell count (provided that CSF is negative for other pathogens by culture or microscopy and negative for malignant cells).
- Disseminated fungal infection:
    - Papular/nodular skin lesions without any other explanation.
    - Intraocular findings suggestive of haematogenous fungal chorioretinitis or endophthalmitis.
- Chronic disseminated candidiasis: Small, peripheral, target-like abscesses ("bull's-eye lesions") in liver and/or spleen demonstrated by CT, magnetic resonance imagery (MRI), or ultrasound, as well as elevated serum alkaline phosphatase. (Note: supporting microbiological criteria are not required.)
- Candidaemia: Clinical criteria are not required for probable candidaemia.

Pharmacokinetic assay methods

PPD Development (Middleton, WI, USA) analysed plasma samples for itraconazole using a validated high-performance liquid chromatography (HPLC) method, which was similar to a previously published method (Srivatsan, Dasgupta *et al*, 2004). The plasma samples (1·00 ml) were processed with liquid phase extraction followed by HPLC separation and fluorescence detection. The dynamic range of the assay for itraconazole was 0·5 to 100 ng/ml.

PPD Development (Richmond, VA, USA) analysed plasma samples for voriconazole using a validated liquid chromatography coupled to tandem-mass spectrometry (LC/MS/MS) method (Andrews, Damle *et al*, 2008). The plasma samples (0·100 ml) were extracted using a solid phase extraction procedure followed by LC/MS/MS separation and detection. The dynamic range of the assay for voriconazole was 10 to 5000 ng/ml.

Further details of statistical analyses

The primary endpoint of success of prophylaxis at day 180 was assessed by calculating the proportion of successes at this time point. For descriptive purposes, the unadjusted success rates for each treatment were presented, along with the difference in proportions, approximate 95% confidence interval (CI) for this difference and corresponding *P* value. The primary analysis involved stratified responder rates, adjusted for both conditioning regimen and relatedness of donor. These two dichotomous factors were combined into a four-level variable representing randomisation strata. The estimated difference between these adjusted success rates and associated 95% CI was

evaluated using the Fleiss method. The resulting CI was used to assess both noninferiority and superiority of voriconazole compared with itraconazole.

Treatment group comparisons of continuous variables were mostly performed using the two-sample *t*-test. *P* values were reported using the pooled variance estimator when the folded F-test for equality of variances was not significant and using Satterthwaite's method when the folded F-test was significant. The Wilcoxon rank-sum test was used to compare both total duration of study medication and duration of liposomal amphotericin B or caspofungin use between treatment groups. A stepwise Cox regression was used to explore the dependent relationship between Treatment Satisfaction Questionnaire for Medication (TSQM) scores and treatment completion status.

One-year survival in each treatment group was summarised with the use of Kaplan-Meier curves, and the difference between treatments was assessed using the log-rank test, with the hazard ratio for death calculated from a Cox regression model with a single treatment covariate.

Reasons for exclusion of screened patients and for not receiving study intervention

Out of 534 patients screened for inclusion into the study, 503 were randomised to study treatment. Reasons for exclusion were not meeting entry criteria (14 patients), adverse events (four patients), HCT not done or postponed (four patients), protocol violations (three patients), lack of clinical supplies at time of randomisation (three patients), residual pulmonary lesion,

patient was moved to different hospital, and death from cerebral haemorrhage (one patient each).

Out of a total of 503 randomised patients, 14 were not treated. Nine patients randomised to voriconazole did not receive treatment, due to not meeting entry criteria (four patients), not taking any study medication throughout the trial (two patients), protocol violations, fungal infection, and previous adverse events with voriconazole (one patient each). In the itraconazole arm, five patients did not receive treatment, due to not meeting entry criteria (two patients), adverse events, lack of clinical supplies at time of enrolment, and not taking any study medication throughout the trial (one patient each).

Information on GvHD and T-cell depletion

Treatment groups were well balanced in the proportion of patients with GvHD overall (46·4% for voriconazole, 44·8% for itraconazole; *P* = 0·73). There were also no differences in GvHD affecting the liver (Supplemental Table I).

Supplemental Table I. Incidence of graft-versus-host disease (GvHD).

Type of GvHD affecting the liver	Voriconazole (*n* = 224) *n* (%)	Itraconazole (*n* = 241) *n* (%)	*P* value for difference
Acute	19 (8·5)	18 (7·5)	*P* = 0·69
Limited chronic*	6 (2·7)	5 (2·1)	*P* = 0·67
Extensive chronic†	0	0	-
Any GvHD affecting the liver	24 (10·7)	22 (9·1)	*P* = 0·57

*Hepatic dysfunction due to chronic GvHD.

†Chronic aggressive hepatitis, bridging necrosis or cirrhosis.

ATG was used after transplant in three voriconazole and three itraconazole patients. One patient in the itraconazole arm took alemtuzumab after study drug discontinuation.

Information on TSQM

TSQM evaluations were only conducted in countries where a validated translation of the questionnaire was available. Sufficient data for a meaningful analysis were only collected at day 14 (see Supplemental Table II). TSQM data were collected for similar proportions of patients in each treatment arm; data were generally missing by site.

Supplemental Table II. Results of treatment satisfaction questionnaire for medication (TSQM).

	Voriconazole (n = 224)	Itraconazole (n = 241)	P value for difference
**TSQM effectiveness score**			
Proportion of patients with data collected, %	61·6	61·8	-
Mean score	74·5 ± 17·6	67·9 ± 19·2	< 0·01
**TSQM side-effects score**			
Proportion of patients with data collected, %	63·4	63·1	-
Mean score	91·7 ± 19·0	88·4 ± 22·6	0·17
**TSQM convenience score**			
Proportion of patients with data collected, %	65·6	64·7	-
Mean score	75·3 ± 16·7	65·0 ± 20·9	< 0·01
**TSQM global satisfaction score**			

Proportion of patients with data collected, %	65·2	63·9	-
Mean score	70·6 ± 15·8	63·1 ± 19·1	< 0·01

Supplemental Table III. Investigator-reported reasons for study drug discontinuation prior to day 100.

Reason for discontinuation	Voriconazole (*n* = 224)	Itraconazole (*n* = 241)
Any reason, *n* (%)	104 (46·4)	147 (61·0)
**Adverse event, *n* (%)**	67 (29·9)	56 (23·2)
Gastrointestinal intolerance	0 (0·0)	12 (5·0)
Hepatic toxicity	38 (17)	10 (4·1)
Other toxicity	9 (4·0)	4 (1·7)
IFI	0	1 (0·4)
Other medical condition*	20 (8·9)	29 (12·1)
**Intolerance, *n* (%)**	15 (6·7)	52 (21·6)
Gastrointestinal intolerance	2 (0·9)	24 (10)
Hepatic toxicity	4 (1·8)	2 (0·8)
Other toxicity	3 (1·3)	1 (0·4)
No specific reason reported	6 (2·7)	24 (10)
Noncompliance, *n* (%)	3 (1·3)	1 (0·4)
Breakthrough IFI, *n* (%)†	1 (0·4)	4 (1·7)
Death, *n* (%)	4 (1·8)	2 (0·8)
No reason given, *n* (%)	2 (0·9)	4 (1·7)
Other, *n* (%)	11 (4·9)	26 (10·8)

*This category comprised a wide variety of other medical conditions, including progression of underlying disease, acute GvHD, fever and/or pneumonia.

†These patients were specifically cited by investigators as having a breakthrough IFI as the reason for treatment discontinuation. Thus, these numbers differ from the incidence of breakthrough IFIs determined by the data review committee.
